# Synthesis, Evaluation of Biological Activity, and Structure–Activity Relationships of New Amidrazone Derivatives Containing Cyclohex-1-ene-1-Carboxylic Acid

**DOI:** 10.3390/molecules30081853

**Published:** 2025-04-21

**Authors:** Renata Paprocka, Jolanta Kutkowska, Ewelina Paczkowska, Godwin Munroe Mwaura, Andrzej Eljaszewicz, Anna Helmin-Basa

**Affiliations:** 1 Department of Organic Chemistry, Faculty of Pharmacy, Collegium Medicum in Bydgoszcz, Nicolaus Copernicus University in Toruń, Jurasza Str. 2, 85-089 Bydgoszcz, Poland; 2 Department of Genetics and Microbiology, Institute of Biological Sciences, Maria Curie-Skłodowska University, Akademicka Str. 19, 20-033 Lublin, Poland; jolanta.kutkowska@mail.umcs.pl; 3 Department of Immunology, Faculty of Pharmacy, Collegium Medicum in Bydgoszcz, Nicolaus Copernicus University in Toruń, M. Curie-Sklodowska Str. 9, 85-094 Bydgoszcz, Polanda.helmin-basa@cm.umk.pl (A.H.-B.); 4 Department of Pharmaceutical Chemistry, Pharmaceutics and Pharmacognosy, Faculty of Health Sciences, University of Nairobi, KNH, Nairobi P.O. Box 2149-00202, Kenya; godwinmunroe1@gmail.com; 5 Centre of Regenerative Medicine, Medical University of Bialystok, Waszyngtona 15 B, 15-269 Bialystok, Poland; andrzej.eljaszewicz@umb.edu.pl

**Keywords:** amidrazones, anti-inflammatory, antiproliferative, antibacterial, cytokines, cyclohexene, structure–activity relationships

## Abstract

In recent years, the incidence of acute and chronic inflammatory diseases has increased significantly worldwide, intensifying the search for new therapeutic agents, especially anti-inflammatory drugs. Therefore, the aim of this work was to synthesize, biologically assess, and explore the structure–activity relationships of new compounds containing the cyclohex-1-ene-1-carboxylic acid moiety. Six new derivatives, **2a**–**2f**, were synthesized through the reaction of amidrazones **1a**–**1f** with 3,4,5,6-tetrahydrophthalic anhydride. Their toxicity was evaluated in cultures of human peripheral blood mononuclear cells (PBMCs). Additionally, their antiproliferative properties and effects on the synthesis of TNF-α, IL-6, IL-10, and IL-1β were assessed in mitogen-stimulated PBMCs. The antimicrobial activity of derivatives **2a**–**2f** was determined by measuring the minimal inhibitory concentration (MIC) values against five bacterial strains—*Staphylococcus aureus*, *Mycobacterium smegmatis*, *Escherichia coli*, *Yersinia enterocolitica*, and *Klebsiella pneumoniae*—and the fungal strain *Candida albicans.* All compounds demonstrated antiproliferative activity, with derivatives **2a**, **2d**, and **2f** at a concentration of 100 µg/mL being more effective than ibuprofen. Compound **2f** strongly inhibited the secretion of TNF-α by approximately 66–81% at all studied doses (10, 50, and 100 µg/mL). Derivative **2b** significantly reduced the release of cytokines, including TNF-α, IL-6, and IL-10, at a high dose (by approximately 92–99%). Compound **2c** exhibited bacteriostatic activity against *S. aureus* and *M. smegmatis*, while derivative **2b** selectively inhibited the growth of *Y. enterocolitica* (MIC = 64 µg/mL). Some structure–activity relationships were established for the studied compounds.

## 1. Introduction

Inflammation refers to the body’s protective response to injury, infection, or harmful stimuli, aiming to eliminate the cause of damage, clear dead cells and debris, and trigger tissue repair. The inflammatory process is usually characterized by redness, swelling, heat, pain, and loss of tissue function, which results from vascular and cellular responses. These complex biological processes are orchestrated by immune cells and the associated inflammatory mediators. In fact, inflammation can be categorized into acute and chronic forms. Acute inflammation is an immediate and transient response caused by the direct disruption of epithelial barriers and involving the recruitment and activation of neutrophils and monocytes/macrophages. These processes are regulated by signals involving pathogen-associated molecular patterns (PAMPs) and danger/damage-associated molecular patterns (DAMPs), leading to the release of inflammatory cytokines, chemokines, and other inflammatory mediators (such as lipid mediators, including prostaglandins and leukotrienes) [1,2,3]. When acute inflammation fails to resolve, it can progress to a chronic process, persisting for months or even years. Chronic inflammation is characterized by the sustained activation of innate and adaptive immune responses involving the recruitment of T cells, B cells, and plasma cells to the affected site [4]. Both acute and chronic inflammation can also be modulated by neutrophil phagocytosis and the release of neutrophil extracellular traps (NETs) [5].

The global prevalence of inflammatory disorders has risen significantly in recent years, intensifying the search for novel therapeutic agents, particularly in the realm of anti-inflammatory drugs [6]. Chronic inflammation underlies a myriad of diseases, including autoimmune disorders, cardiovascular diseases, and cancer, necessitating ongoing research into more effective treatments. While current anti-inflammatory drugs demonstrate efficacy, they often come with severe side effects that limit their long-term use. This limitation has spurred the development of new agents [7]. Continuous research and development in this field aim to provide novel solutions to combat inflammatory diseases and improve the quality of life for affected individuals globally.

Amidrazone derivatives have emerged as promising candidates in drug discovery due to their diverse biological activities and structural versatility. These compounds have demonstrated notable anti-inflammatory properties, with studies showing their ability to modulate key cytokines such as IL-6, IL-10, and IL-1β, and tumor necrosis factor-alpha (TNF-α), which are critical mediators in inflammatory responses [8]. Specifically, triazole derivatives have exhibited strong anti-inflammatory effects through this mechanism [9]. Certain derivatives have also displayed antiproliferative activity in mitogen-activated peripheral blood mononuclear cell (PBMC) cultures, indicating their potential in controlling cell proliferation [10]. These effects highlight their broader therapeutic utility beyond inflammation management [10].

The significance of amidrazone derivatives in medicinal chemistry is underscored by their wide range of biological activities. They have demonstrated potential as tuberculostatic, antibacterial, antifungal, antiparasitic, antiviral, cytoprotective, and antitumor agents, as well as being inhibitors of furin and acetylcholinesterase [8,11].

Bioactive compounds containing the cyclohexene moiety can be isolated from raw materials of natural origin. For example, diisoprenylcyclohexene-type meroterpenoids isolated from *Biscogniauxia* sp. exhibited anti-inflammatory effects by inhibiting the production of pro-inflammatory cytokines TNF-α and IL-6 [12]. Among the polyoxygenated cyclohexene derivatives isolated from the stem of *Uvaria rufa*, 6-acetylzeylenol demonstrated activity against *Mycobacterium tuberculosis* [13]. The anti-inflammatory effect of (-)-zeylenone, isolated from *Uvaria macclurei*, has been demonstrated through the reduction in nitric oxide (NO) synthesis in mitogen-stimulated RAW 264.7 macrophages derived from mice and the inhibition of NF-κB signaling pathway activation. Additionally, it exhibited anti-sepsis activity in mice [14]. A synthetic analogue of zeylenone also showed strong antiproliferative activity in glioblastoma cells [15].

In our previous work, compounds derived from amidrazones containing the cyclohex-1-ane-1-carboxylic acid moiety demonstrated various biological activities, including antiviral, antibacterial, anti-inflammatory, and antinociceptive properties [16]. Encouraged by these results, we designed new amidrazone derivatives possessing an unsaturated cyclohex-1-ene-1-carboxylic acid system to evaluate their basic biological effects, such as toxicity and antiproliferative, anti-inflammatory, and antimicrobial activities. Additionally, we aim to investigate the influence of the double bond in the cyclohexene system and the type of substituents present in the synthesized compounds on their biological activity.

## 2. Results

### 2.1. The Synthesis of Compounds ***2a**–**2f***

**Scheme 1:**
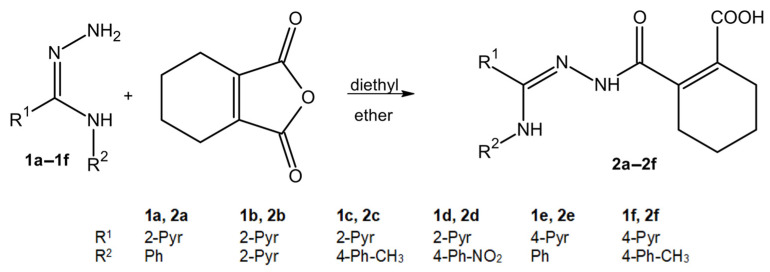
The synthesis of compounds **2a**–**2f** from the reaction of amidrazones **1a**–**1f** with 3,4,5,6-tetrahydrophthalic anhydride.

The reactions of amidrazones **1a**–**1f** with 3,4,5,6-tetrahydrophthalic anhydride yielded six new acyl derivatives, **2a**–**2f** (Scheme 1). All reactions proceeded with very high yields (above 90%), except for derivative **2d**, which was synthesized from amidrazone containing a 4-nitrophenyl substituent at the R^2^ position. This substituent may have hindered the reaction.

In the ^1^H NMR and ^13^C NMR spectra of compounds **2a**–**2f**, a double number of signals is observed, consistent with the occurrence of E/Z isomerism around the C=N bond. This phenomenon has been previously described in acylamidrazones [17]. Elemental analysis of the synthesized compounds **2a**–**2f**, along with HRMS analysis, confirmed their elemental composition.

### 2.2. Toxicity of Compounds ***2a**–**2f***

**Figure 1:**
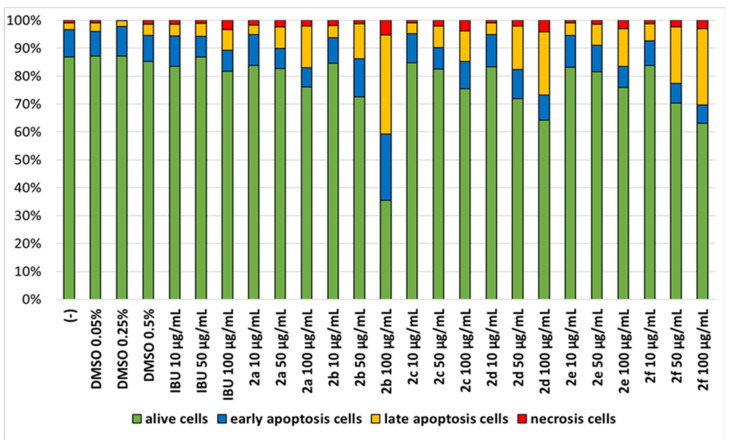
Frequencies of viable, early apoptotic, late apoptotic, and necrotic cells in PBMC cultures (24 h) with compounds **2a**–**2f** and IBU at concentrations of 10, 50, and 100 µg/mL.

First, we aimed to assess the toxic effects of the new derivatives. The data from the toxicity assessments of compounds **2a**–**2f** and the reference anti-inflammatory agent, ibuprofen (IBU), on peripheral blood mononuclear cells (PBMCs) in 24 h in vitro cultures are presented in Figure 1. PBMC cultures incubated with a vehicle, namely dimethyl sulfoxide (DMSO) at concentrations of 0.05–0.50%, served as a control. As expected, approximately 86% of viable cells were observed in control cultures, with about 10% of cells in early apoptosis. In cultures with IBU at a concentration of 100 µg/mL, approximately 7.5% of early apoptotic and 7.5% of late apoptotic cells were observed. In PBMC cultures incubated with derivatives **2a**, **2c**, and **2e** at the highest concentration, 100 µg/mL, approximately 76% of viable cells were present, and the level of late apoptotic cells was slightly elevated (approximately 11–15%).

Compound **2b** showed strong dose-dependent toxicity, while derivatives **2d** and **2f** exhibited moderate dose-dependent toxicity. At a concentration of 100 µg/mL, compound **2b** caused the highest frequencies of early apoptotic (23.66%), late apoptotic (35.51%), and necrotic (5.31%) cells compared to other PBMC cultures. In PBMC cultures with compounds **2d** and **2f** at a concentration of 100 µg/mL, approximately 6–9% of early apoptotic cells, 22.5% and 27% of late apoptotic cells, and 3–4% of necrotic cells were present. At the highest culture dose, the percentage of necrotic cells for individual compounds **2a**–**2f** was comparable to the percentage of necrotic cells in cultures containing IBU at the same dose.

### 2.3. Antiproliferative Activity of Compounds ***2a**–**2f***

**Figure 2:**
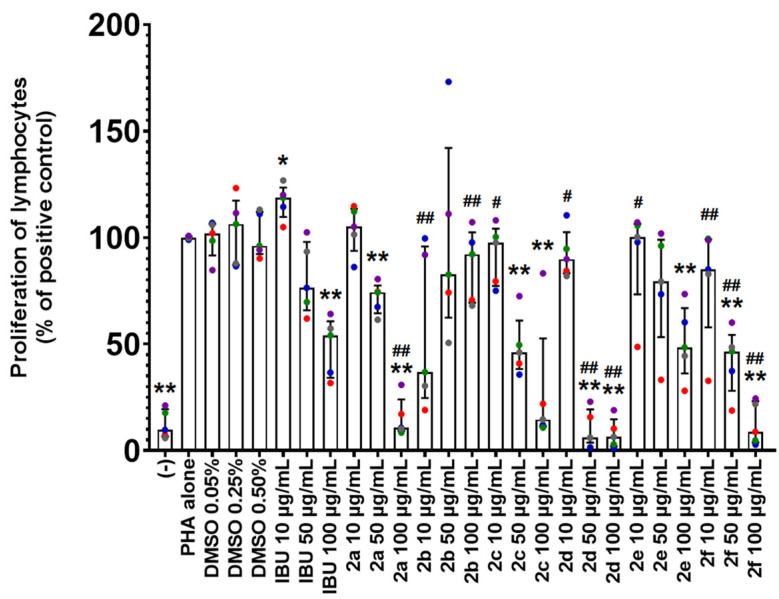
The antiproliferative effect of compounds **2a**–**2f** on a 24 h PBMC culture stimulated with phytohemagglutinin (PHA). Results (from 5 independent experiments, expressed as median and Q1–Q3 range) are presented as a percentage of the positive control (cultures stimulated with PHA alone). Dots in different colors indicate the results of independent experiments. The symbols indicate the statistical significance (Mann–Whitney U test): * vs. DMSO, *p* < 0.05; # vs. IBU, *p* < 0.05; ** vs. DMSO *p* < 0.01; ## vs. IBU, *p* < 0.01.

Having found no cytotoxic effects of the analyzed compounds, we next aimed to evaluate their impact on mitogen-induced PBMC proliferation. Again, PBMCs incubated in the presence of the vehicle (DMSO at the same concentrations as in cultures with the tested compounds) served as a control. No significant effect of the solvent on phytohemagglutinin (PHA)-stimulated T lymphocyte proliferation was observed (Figure 2). IBU at a dose of 10 µg/mL slightly increased the proliferation of T lymphocytes. However, at a concentration of 100 µg/mL, significant T cell inhibition (approximately 50%) was observed in the cultures. Compounds **2a**, **2c**, **2e**, **2f**, and IBU showed dose-dependent antiproliferative activity. The highest inhibition (approximately 95%) was observed for compound **2d** at concentrations of 50 µg/mL and 100 µg/mL, as well as for derivatives **2a** and **2f** at a concentration of 100 µg/mL (approximately 90% inhibition). Among derivatives **2a**–**2f**, at a dose of 100 µg/mL, only compound **2b** did not decrease the proliferation of T cells. However, compound **2b** was the only one that inhibited T lymphocyte proliferation by approximately half at the lowest dose of 10 µg/mL.

### 2.4. Effect of Compounds ***2a**–**2f*** on TNF-α Production

**Figure 3:**
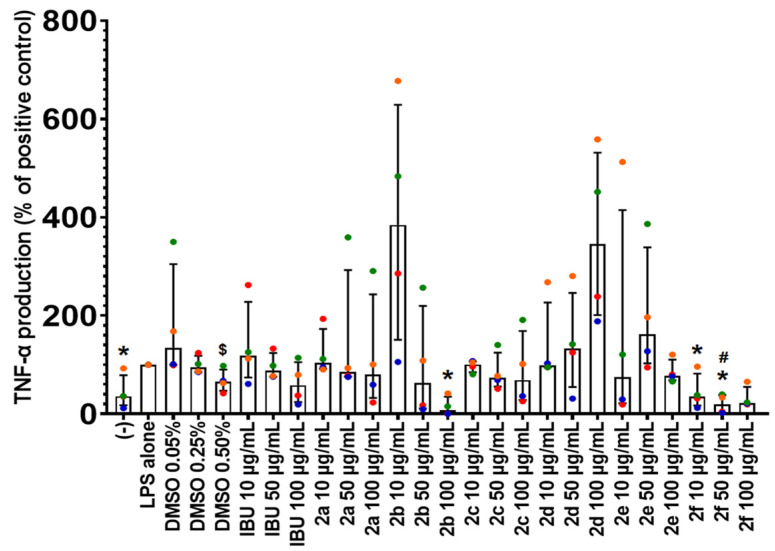
The effect of compounds **2a**–**2f** on TNF-α production in a 72 h PBMC culture stimulated with lipopolysaccharide (LPS). Results (from 4 independent experiments, expressed as median and Q1–Q3 range) are presented as a percentage of the positive control (cultures stimulated with LPS alone). Dots in different colors indicate the results of independent experiments. The symbols indicate the statistical significance (Mann–Whitney U test): * vs. DMSO, *p* < 0.05; # vs. IBU, *p* < 0.05; $ vs. LPS alone, *p* < 0.05.

Next, we assessed the effects of the analyzed compounds on the pro-inflammatory TNF-α production of endotoxin-exposed PBMCs. DMSO at the highest concentration of 100 µg/mL caused a significant reduction in TNF-α production in an LPS-stimulated PBMC culture (Figure 3). Both derivatives—**2f** at concentrations of 10 µg/mL and 50 µg/mL, and **2b** at a concentration of 100 µg/mL—showed significant inhibition of TNF-α production.

### 2.5. Effect of Compounds ***2a**–**2f*** on IL-6 Production

**Figure 4:**
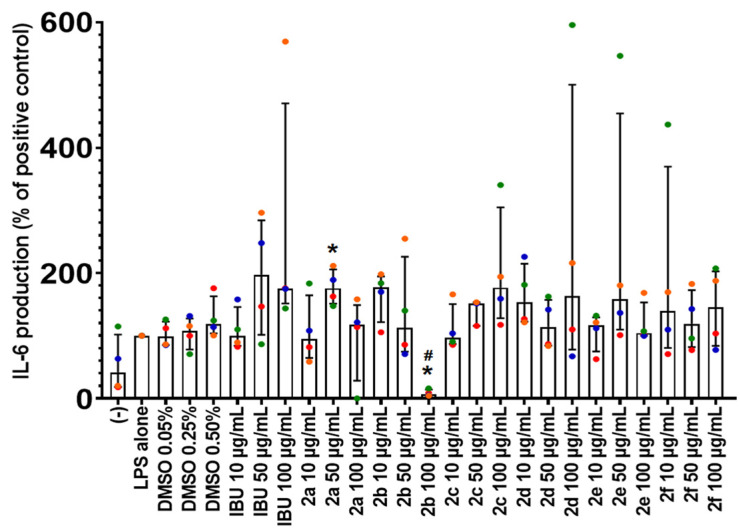
The effect of compounds **2a**–**2f** on IL-6 production in a 72 h PBMC culture stimulated with lipopolysaccharide (LPS). Results (from 4 independent experiments, expressed as median and Q1–Q3 range) are presented as a percentage of the positive control (cultures stimulated with LPS alone). Dots in different colors indicate the results of independent experiments. The symbols indicate the statistical significance (Mann–Whitney U test): * vs. DMSO, *p* < 0.05; # vs. IBU, *p* < 0.05.

Having found changes in TNF-α production, we next assessed the effects on IL-6 release. Derivative **2b** at a dose of 100 µg/mL showed strong inhibition of the secretion of this pro-inflammatory cytokine (Figure 4). However, compound **2a** at a concentration of 50 µg/mL significantly increased IL-6 production in an LPS-stimulated PBMC culture. No significant changes in IL-6 concentration were observed in the remaining cultures (*p* > 0.05).

### 2.6. Effect of Compounds ***2a**–**2f***on IL-10 Production

**Figure 5:**
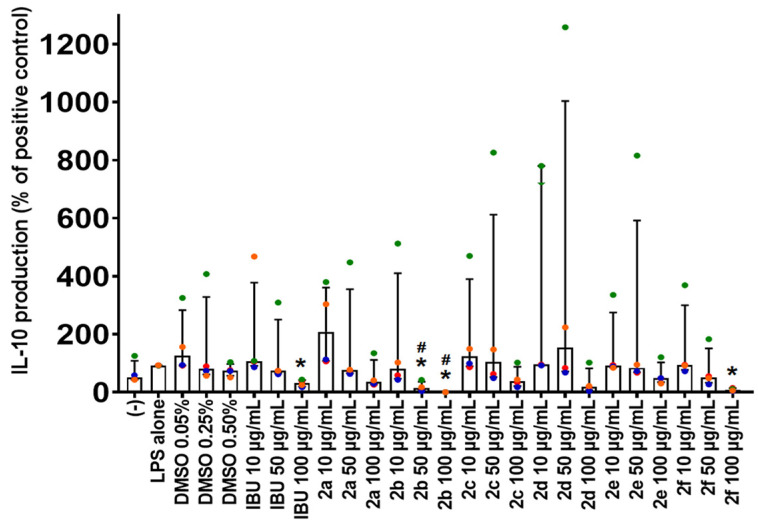
The effect of compounds **2a**–**2f** on IL-10 production in a 72 h PBMC culture stimulated with lipopolysaccharide (LPS). Results (from 4 independent experiments, expressed as median and Q1–Q3 range) are presented as a percentage of the positive control (cultures stimulated with LPS alone). Dots in different colors indicate the results of independent experiments. The symbols indicate the statistical significance (Mann–Whitney U test): * vs. DMSO, *p* < 0.05; # vs. IBU, *p* < 0.05.

Finally, we assessed the effect of the analyzed compounds on anti-inflammatory IL-10 production in the PBMC response to LPS. A significant reduction in the release of the IL-10 cytokine was observed in the case of the following compounds: IBU (at a concentration of 100 µg/mL), **2b** (at concentrations of 50 and 100 µg/mL), and **2f** (at a concentration of 100 µg/mL, Figure 5). The remaining compounds did not show significant differences in IL-10 levels compared to the LPS-stimulated culture.

### 2.7. The Influence of Compounds ***2a**–**2f***on IL-1β Production

No significant changes in the level of IL-1β were observed for any of the studied compounds (*p* > 0.05, Figure S15 in the Supplementary Materials).

### 2.8. Antimicrobial Activity of Compounds ***2a**–**2f***

The tested derivatives were found to possess moderate or weak activity against the tested strains; MIC values ranged from 64 to greater than 512 µg/mL (Table 1). The best anti-mycobacterial activity was shown by compounds **2a** and **2c**, which inhibited *M. smegmatis* growth at a concentration of 64 µg/mL. Moreover, they were effective against *S. aureus* at concentrations of 64 µg/mL (derivative **2c**) and 256 µg/mL (derivative **2a**).

Derivatives **2b** and **2f** showed moderate activity against *Y. enterocolitica,* with MIC values of 64 µg/mL and 128 µg/mL, respectively. Derivative **2b** also inhibited the growth of *E. coli* and *K. pneumoniae* at a concentration of 256 µg/mL. Compound **2f** revealed weak antifungal activity and inhibited the growth of *C. albicans* at a concentration of 256 µg/mL. The remaining compounds, **2d** and **2e**, did not exhibit antimicrobial activity, with MIC values of ≥512 µg/mL.

Four of the bacterial strains tested were sensitive to ampicillin, with MIC values of 0.5 and 8 µg/mL for *S. aureus* and *E. coli* and 16 µg/mL for *Y. enterocolitica* and *M. smegmatis*. *K. pneumoniae* was resistant to ampicillin (MIC > 256 µg/mL), whereas *C. albicans* was sensitive to fluconazole (MIC 0.25 µg/mL).

## 3. Discussion

**Figure 6:**
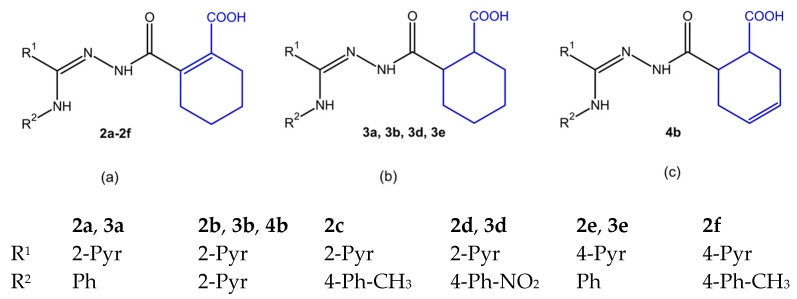
Amidrazone derivatives **2a**–**2f** containing (**a**) cyclohex-1-ene-1-carboxylic acid moiety, (**b**) derivatives **3a**, **3b**, **3d**, and **3e** with cyclohexanecarboxylic acid moiety, or (**c**) **4b** with cyclohex-3-ene-1-carboxylic acid moiety, as described in previous works [16,18].

Six new acyl derivatives, **2a**–**2f**, were easily synthesized in a reaction of *N*^3^-substituted amidrazones with 3,4,5,6-tetrahydrophthalic anhydride in anhydrous diethyl ether (Figure 6a).

Among closely related compounds, only a few amidrazone derivatives—**3a**, **3b**, **3d**, and **3e**—containing the cyclohexanecarboxylic acid moiety (Figure 6b) [16] and only one derivative, **4b**, containing the cyclohex-3-ene-1-carboxylic acid system (Figure 6c) [18], have been described. These compounds showed a wide range of biological activities and high potency, including antinociceptive and anti-inflammatory effects. This encouraged us to synthesize and investigate the biological activity of analogous compounds, **2a**–**2f**, containing an additional double bond in the aliphatic ring (cyclohex-1-ene-1-carboxylic acid moiety).

Six new acyl derivatives, **2a**–**2f**, were easily synthesized in a reaction of *N*^3^-substituted amidrazones with 3,4,5,6-tetrahydrophthalic anhydride in anhydrous diethyl ether. In previously described reactions, it was not possible to obtain the expected acyclic compounds, **3c** and **3f**, containing a 4-methylphenyl substituent in the R^2^ position, probably due to their tendency to undergo cyclisation to 1,2,4-triazole derivatives [16]. The successful synthesis of derivatives **2c** and **2f** in the current approach was facilitated, likely due to the shortening of the reaction time from 14 to 3 days or due to the more favorable properties of 3,4,5,6-tetrahydrophthalic anhydride compared to *cis*-1,2-cyclohexanedicarboxylic anhydride.

The next step in this work was the assessment of the toxicity of six new derivatives. Flow cytometric detection of apoptosis in PBMC culture showed toxicity for derivatives **2b**, **2d**, and **2f** at a concentration of 100 µg/mL (about 35%, 64%, and 63% of viable cells, respectively). However, these compounds at concentrations of 10 and 50 µg/mL, as well as compounds **2a**, **2c**, and **2e**, and IBU at concentrations of 10 µg/mL, 50 µg/mL, and 100 µg/mL, showed satisfactory cell survival in the range of 70–85%. For comparison, the previously studied compound **4b**, having a double bond on the opposite side of the cyclohexene ring, was not significantly toxic to PBMCs in the concentration range of 1–100 µg/mL [18]. In turn, compound **3b**, possessing a saturated cyclohexane ring, was studied in a human fibroblast CCD-18C0 cell culture and showed high toxicity at concentrations of 50 µg/mL and 100 µg/mL (about 35 and 24% of viable cells, respectively) [16].

Due to the small number of tested compounds and the use of different research models (fibroblast or PBMC), it is difficult to clearly indicate which structural element of these derivatives influenced cellular toxicity. It can be assumed that the presence of two 2-pyridyl substituents increases the toxicity of compounds **2b** and **3b**, while the presence of a cyclohex-3-ene-1-carboxylic acid moiety in derivative **4b** reduces this effect.

The subsequent step of this work was to determine the antiproliferative effects of compounds **2a**–**2f** on PHA-stimulated lymphocytes. We showed that the inhibitory effect of compounds **2a**–**2f** depended on the type of their substituents, R^1^ and R^2^. Moreover, derivatives **2a**, **2c**, **2e**, and **2f** and IBU showed a dose-dependent inhibitory effect. In contrast, compound **2b** caused about 50% inhibition of lymphocyte proliferation at the lowest dose, 10 µg/mL, but was ineffective at the other doses. For the most active compound, **2d**, at a dose of 50 µg/mL, and for compounds **2a**, **2d**, and **2f** at a dose of 100 µg/mL, showing about 90–95% inhibitory effect, the IBU activity (about 46%) was significantly exceeded. These results are consistent with the high toxicity of derivatives **2d** and **2f**. However, compounds **2a**, **2c**, **2e**, and IBU were not toxic at a concentration of 100 µg/mL, so their observed dose-dependent antiproliferative effect was not associated with toxicity but rather with their anti-inflammatory effect.

**Figure 7:**
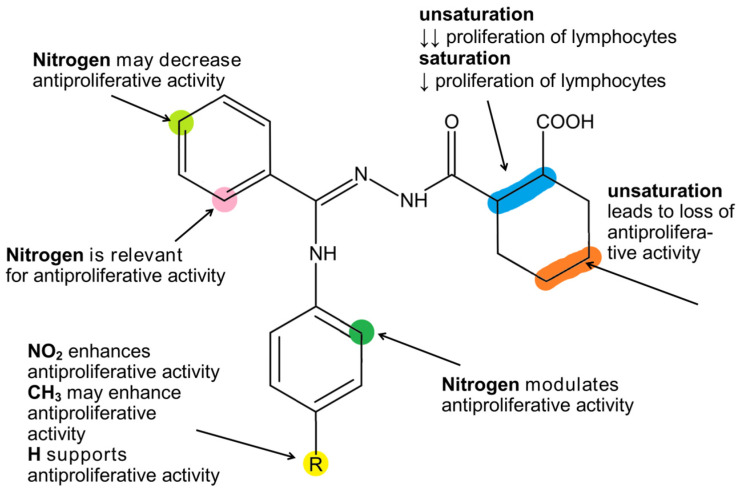
Structure–antiproliferative activity relationships observed for amidrazone derivatives **2a**–**2f**, **3a**, **3b**, **3d**, **3e**, and **4b**.

Among the analogous compounds **3a**, **3b**, **3d**, **3e**, and **4d**, also studied in PHA-stimulated PBMC cultures at a dose of 100 µg/mL, the strongest antiproliferative effect was observed for compound **3b**, possessing two 2-pyridyl substituents (approximately 61% inhibition). Weaker activity was observed for compounds **3d** and **3e** (approximately 28% inhibition) and **3a** (approximately 12% inhibition) [16]. No antiproliferative activity was detected for derivative **4b** [18]. The presence of a double bond in the cyclohexene ring increased the antiproliferative activity of compounds **2a** and **2e** (which have a phenyl ring in the R^2^ position) and **2d** (which has a 4-nitrophenyl substituent in the R^2^ position) in comparison to the cyclohexane derivatives **3a**, **3d**, and **3e**. In the case of compounds **2b**, **3b**, and **4b**, containing two 2-pyridyl rings in the R^2^ position, antiproliferative activity varied depending on their structures and the dose used (Figure 7).

To investigate the anti-inflammatory activity of compounds **2a**–**2f**, we also examined their effect on pro-inflammatory cytokine (IL-6, TNF-α, IL-1β) levels in LPS-stimulated PBMC cultures. Among them, only compound **2b** showed a significant decrease in IL-6 secretion at the highest dose of 100 µg/mL (approximately 93% inhibition). In a previous study, we also observed a weak IL-6 inhibitory effect of compound **3b** (approximately 10–12%) and a stronger effect for **3a** (approximately 35%) at a concentration of 10 µg/mL [16].

A more complex situation occurred when examining the influence of compounds **2a**–**2f** on the secretion of TNF-α. Derivative **2f** decreased the TNF-α level at all studied doses (by approximately 66–81%). Compound **2b** was significantly inhibitory at a concentration of 100 µg/mL (approximately 92%). Only derivative **2d** significantly increased the level of TNF-α at a concentration of 100 µg/mL. Analogous compounds **3a**, **3b**, **3d**, **3e**, and **4b**, studied at a concentration of 10 µg/mL, caused TNF-α inhibition of approximately 40%.

The presence of a double bond in the cyclohexene ring significantly reduced the inhibitory effect of compounds **2a**, **2b**, **2d**, and **2e** at a concentration of 10 µg/mL on TNF-α production compared to derivatives **3a**, **3b**, **3d**, and **3e**, although it did not change the activity of compound **4b** (having a double bond on the other side of the cyclohexane ring) compared to **3b**.

**Figure 8:**
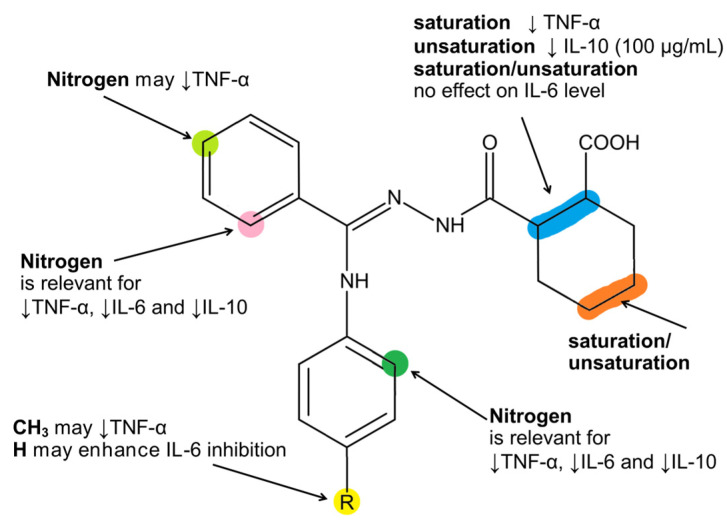
Structural elements of amidrazone derivatives **2a**–**2f**, **3a**, **3b**, **3d**, **3e**, and **4b** and their impact on the levels of IL-6, TNF-α, and IL-10 cytokines.

We also studied the effect of compounds **2a**–**2f** on the secretion of the anti-inflammatory cytokine IL-10 in LPS-stimulated PBMC cultures. An inhibitory effect on IL-10 release was observed at the highest dose for compounds **2f** and IBU, as well as for compound **2b** at medium and the highest doses (Figure 5). All compounds, **2a**–**2f**, showed a tendency to inhibit IL-10 at a dose of 100 µg/mL, but only for compounds **2b** and **2f** was this effect significant. A summary of the effects of the studied compounds on cytokine levels is presented in Figure 8.

In the second part of the biological tests, the antimicrobial activity of compounds **2a**–**2f** was examined. The most active antibacterial compounds possessed a 2-pyridyl substituent in the R^1^ position (compounds **2a**–**2c**). The exception was compound **2d**, containing a 4-nitrophenyl substituent in the R^2^ position, which was devoid of any antimicrobial activity. Compound **2b**, containing two 2-pyridyl rings, showed selective activity against the Gram-negative strain of *Y. enterocolitica*. The presence of a 2-pyridyl in the R^1^ position and a phenyl (or 4-methylphenyl) substituent in the R^2^ position appears to have a beneficial effect on the antituberculosic activity of compounds **2a** and **2c** against *M. smegmatis*.

In a previous study, we also observed an enhancement of the antibacterial activity for compounds possessing the 4-methylphenyl group in the R^2^ position. For example, derivative **2c** showed stronger activity than **2a** against *S. aureus* and *K. pneumoniae* [16]. Similarly, the enhanced activity of compound **2f** compared to **2e** against *Y. enterocolitica* and *C. albicans* was also observed [16].

Compared to the previously described analogous compounds **3a**, **3b**, and **3e**, adding a double bond to the cyclohexane ring resulted in the following changes in biological activity:

(1) Increased activity of compound **2b** against *Y. enterocolitica*;
(2) Reduced bacteriostatic effect of compound **2a** against *S. aureus*;
(3) Reduced bacteriostatic effect of compound **2e** against *M. smegmatis*.

**Figure 9:**
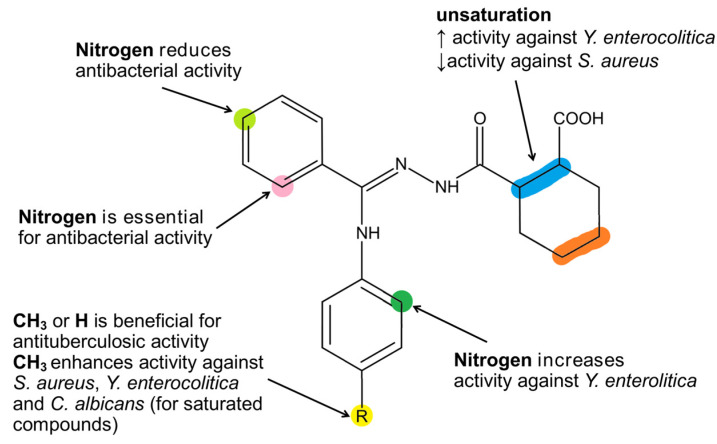
Structure–antimicrobial activity relationships observed for amidrazone derivatives **2a**–**2f**, **3a**, **3b**, **3d**, and **3e**.

A summary of these dependencies is presented in Figure 9.

The structure–activity relationships in Figure 7, Figure 8 and Figure 9 can be summarized in the following conclusions:

The 2-pyridyl substituent in the R^1^ position is crucial for the antiproliferative activity of the studied amidrazone derivatives. Moreover, 4-nitrophenyl or 4-methylphenyl substituents in the R^2^ position can enhance this activity. The presence of a double bond in the cyclohex-1-ene ring further enhances the antiproliferative activity.

The presence of two pyridyl substituents in the R^1^ and R^2^ positions is the most effective for the inhibition of cytokines (TNF-α, IL-6, IL-10). In general, saturation of the cyclohexane ring enhances the inhibitory effect of the compounds on the TNF-α level (except for compound **3b**).

The 2-pyridyl substituent in the R^1^ position is crucial for the antibacterial activity of the studied compounds. The 4-CH_3_-phenyl substituent in the R^2^ position is beneficial for enhancing both antituberculosic and antibacterial activity, while the 4-nitrophenyl substituent is detrimental to antimicrobial activity. Saturation of the cyclohexene bond may alter activity.

The presented relationships have certain limitations related to the small number of substituents considered. Our future research aims to expand this scope to include halogen atoms, methoxy groups, and other functional groups, enabling a broader understanding of the relationship between structure and biological activity in acylamidrazone derivatives.

## 4. Materials and Methods

### 4.1. General

Reagents and solvents were acquired from Sigma-Aldrich or Avantor Performance Materials Poland. Melting points were measured using a Mel-Temp apparatus (Electrothermal). ^1^H NMR and ^13^C NMR analyses were performed in deuterated dimethyl sulfoxide (DMSO-d_6_) on a Bruker Avance III Spectrometer (700 MHz and 400 MHz) and on a Bruker Avance (300 MHz) Spectrometer (Bruker Corporation, Billerica, MA, USA). Elemental analysis was carried out using a Vario MACRO CHN ELEMENTAR (Analysensysteme GmbH, Langenselbold, Germany) elemental analyzer. HRMS (high-resolution mass spectrometry) was recorded on a Synapt G2-S*i* mass spectrometer (Waters Corporation, Milford, MA, USA) equipped with an ESI source and a quadrupole–time–of–flight mass analyzer. The results were analyzed using MassLynx 4.1 software (Waters Corporation, Milford, MA, USA). The course of the reactions was monitored using TLC chromatography (silica gel on TLC-PET foils, Sigma-Aldrich, St. Louis, MO, USA).

### 4.2. General Method for the Synthesis of Compounds ***2a**–**2f***

The amidrazones **1a**–**1f** required for the synthesis were obtained by reacting thioamides with 64% hydrazine hydrate [19]. In general, 0.3 g of amidrazones **1a**–**1f** (about 0.9 mmol) and 0.2 g of 3,4,5,6-tetrahydrophthalic anhydride (approximately 0.9 mmol) were dissolved in 30 mL of diethyl ether. After 3 days, the obtained solid compounds **2a**–**2f** were filtered and washed with 10 mL of diethyl ether to remove unreacted substrates, and dried.

*2-[(2Z)-2-{phenyl[(pyridin-2-yl)amino]methylidene}hydrazinecarbonyl]cyclohex-1-ene-1-carboxylic acid* (**2a**)

m.p. 119–120 °C, yield 92.5%. ^1^H NMR (700 MHz, DMSO-d_6_) δ 12.40 (s, 1 H, COOH). 10.06 (s, 0.48H), 9.77 (s, 0.52H), 8.81–6.79 (m, 9H + 1NH), 6.61 (d, 2H, CH=), 2.36–2.08 (m, 4H), 1.69–1.50 (m, 4H) ppm. ^13^C NMR (75 MHz, DMSO-d_6_): 172.58, 172.02, 168.49, 168.10, 167.68, 166.47, 152.63, 149.29, 148.93, 148.79, 144.77, 144.76, 140.64, 138.74, 137.50, 137.30, 130.88, 130.11, 129.47, 129.37, 129.10, 126.30, 124.63, 123.78, 122.54, 120.50, 119.22, 118.93, 28.57, 28.40, 25.59, 25.04, 22.02, 21.77, 21.64, 21.54, 20.84, 20.68, 15.63 ppm. Elem. Anal. for C_20_H_20_N_4_O_3_: calculated, C, 65.92, H, 5.53, N, 15.38%; found, C, 66.01%, H, 5.70%, N, 15.21%. HR-MS *m*/*z* 363.1463 [M^+^ − 1] (calculated for C_20_H_19_N_4_O_3_: 363.1457).

*2-[(2Z)-2-{(pyridin-2-yl)[(pyridin-2-yl)amino]methylidene}hydrazinecarbonyl]cyclohex-1-ene-1-carboxylic acid* (**2b**)

m.p. 133–135 °C, yield 94.16%. ^1^H NMR (400 MHz, DMSO-d_6_): 12.41 (sb, 1H), 11.93 (s, 0.58H), 11.18 (s, 0.42H), 9.27 (d, 1H NH), 8.49 (d, 1H), 8.15–7.33 (m, 6 H), 7.07–6.81 (m, 2H, CH=), 2.30 (s, 4 H), 1.66 (d, 42), 1.60 (s, 2H) ppm. ^13^C NMR (100 MHz, DMSO-d_6_): 172.86, 168.63, 167.95, 166.50, 154.25, 153.94, 152.56, 152.39, 148.38, 147.25, 146.93, 144.71, 142.13, 140.93, 139.13, 138.78, 137.88, 137.56, 129.92, 126.44, 124.78, 124.46, 122.60, 121.84, 116.94, 116.60, 113.29, 112.72, 65.33, 28.45, 28.30, 25.74, 25.07, 22.07, 21.79, 21.61, 20.81, 20.61 ppm. Elem. Anal. for C_19_H_19_N_5_O_3_: calculated, C, 62.46, H, 5.24, N, 19.17%; found, C, 62.45%, H, 5.37%, N, 19.18%. HR-MS *m*/*z* 364.1419 [M^+^ − 1] (calculated for C_19_H_18_N_5_O_3_: 364.1410).

*2-{(2Z)-2-[(4-methylanilino)(pyridin-2-yl)methylidene]hydrazinecarbonyl}cyclohex-1-ene-1-carboxylic acid* (**2c**)

m.p. 114–118 °C, yield 91%. ^1^H NMR (300 MHz, DMSO-d_6_): 12.30 (sb, COOH), 9.98 (s, 0.48H), 9.62 (s, 0.52H), 8.60–6.87 (m, 8H + 1NH), 6.53 (d, CH=), 2.40–2.05 (m, 9H), 1.73–1.48 (m, 4H) ppm. ^13^C NMR (75 MHz, DMSO-d_6_): 172.57, 172.02, 168.49, 168.10, 167.69, 166.48, 152.88, 152.62, 149.30, 148.92, 148.81, 144.78, 143.11, 140.64, 138.72, 138.66, 137.46, 137.29, 129.44.129.38, 129.12, 126.30, 124.73, 124.63, 124.57, 123.80, 122.53, 120.50, 119.22, 118.93, 65.38, 28.57, 28.40, 25.60, 25.02, 22.03, 21.74, 21.63, 21.54, 20.84, 20.68, 15.6389 ppm. Elem. Anal. for C_21_H_22_N_4_O_3_: calculated, C, 65.66, H, 5.86, N, 14.81%; found, C, 66.22, H, 5.89, N, 14.77%. HR-MS *m*/*z* 377.1619 [M^+^ − 1] (calculated for C_21_H_21_N_4_O_3_: 377.1614).

*2-[(2Z)-2-{[(5-nitropyridin-2-yl)amino](pyridin-2-yl)methylidene}hydrazinecarbonyl]-cyclohex-1-ene-1-carboxylic acid* (**2d**)

m.p. 144–145 °C, yield 57.41%. ^1^H NMR (400 MHz, DMSO-d_6_) 12.45 (s, 1H, COOH), 10.58 (s, 0.37H, CONH), 10.46 (s, 0.63H, CONH), 9.28 (d, 1H, NH), 8.20–7.58 (m, 6H), 7.50–7.38 (m, 1H), 6.76–6.63 (m, 2H, CH=), 2.41–1.15 (m, 4H), 1.68 (s, 2H), 1.56 (s, 2H) ppm. ^13^C NMR (100 MHz, DMSO-d_6_): 173.19, 172.50, 168.11, 167.66, 167.45, 152.04, 149.49, 149.20, 149.05, 145.37, 144.91, 140.60, 140.25, 139.72, 138.88, 138.28, 137.74, 126.32, 125.43, 125.14, 124.96, 124.84, 123.08, 122.02, 117.76, 117.15, 65.43, 116.58, 28.74, 28.47, 25.42, 25.00, 22.02, 21.92, 21.68, 21.46 ppm. Elem. Anal. for C_20_H_19_N_5_O_5_: calculated, C, 58.68, H, 4.68, N, 17.11%; found, C, 58.64, H, 4.60, N, 16.86%. HR-MS *m*/*z* 408.1313 [M^+^ − 1] (calculated for C_20_H_18_N_5_O_5_: 408.1308).

*2-{(2Z)-2-[anilino(pyridin-4-yl)methylidene]hydrazinecarbonyl}cyclohex-1-ene-1-carboxylic acid* (**2e**)

m.p. 118–120 °C, yield 94.20%. ^1^H NMR (700 MHz, DMSO-d_6_) 12.40 (s, 1 H, COOH), 10.05 (s, 0.48H), 9.77 (s, 0.52H), 8.82–6.80 (9H arom + 1HN), 6.61 (d, CH=), 3.35–2.08 (m, 4H), 1.69–1.50 (m, 4H) ppm. ^13^C NMR (176 MHz, DMSO-d_6_): 173.13, 168.44, 168.35, 168.03, 167.59, 150.35, 150.26, 150.18, 144.54, 142.29, 141.55, 141.29, 140.41, 139.13, 129.36, 129.34, 129.05, 126.56, 124.74, 124.00, 123.71, 123.08, 122.35, 122.30, 121.46, 120.98, 120.45, 118.90, 28.69, 28.41, 25.53, 25.02, 22.00, 21.76, 21.64, 21.51, 21.33, 21.12, 19.98, 19.87 ppm. Elem. Anal. for C_20_H_20_N_4_O_3_: calculated, C, 65.92, H, 5.53, N, 15.38%; found, C, 66.00%, H, 5.61%, N, 15.02%. HR-MS *m*/*z* 363.1464 [M^+^ − 1] (calculated for C_20_H_19_N_4_O_3_: 363.1457).

*2-{(2Z)-2-[(4-methylanilino)(pyridin-4-yl)methylidene]hydrazinecarbonyl}cyclohex-1-ene-1-carboxylic acid* (**2f**)

m.p. 132–134 °C, yield 90.00%. ^1^H NMR (400 MHz, DMSO-d_6_): 12.30 (sb, 1 H, COOH), 10.30 (s, 0.5 H, NHCO), 10.24 (s, 0.5 H, NHCO), 8.80–6.92 (m, 8 H + 1NH), 6.57 (d, 1H CH=), 6.50 (d, 1H CH=), 2.34–2.21 (m, 4 H), 2.18 (s, 3 H), 1.64 (s, 2H), 1.61 (s, 2H) ppm. ^13^C NMR (100 MHz, DMSO-d_6_): 173.05, 168.48, 168.38, 168.03, 166.84, 150.18, 150.09, 144.56, 143.28, 142.43, 142.36, 140.35, 139.56, 131.63, 130.65, 129.82, 129.44, 128.70, 126.47, 124.32, 123.69, 123.18, 122.43, 122.30, 121.07, 119.55, 28.71, 28.38, 25.54, 25.03, 21.99, 21.76, 21.63, 21.53, 20.92, 20.80, 20.67, 19.99, 19.84 ppm. Elem. Anal. for C_21_H_22_N_4_O_3_: calculated, C, 66.65%; H, 5.86%; N, 14.75%; found, C, 66.82%; H, 5.89%; N, 14.81%. HR-MS *m*/*z* 377.1620 [M^+^ − 1] (calculated for C_21_H_21_N_4_O_3_: 377.1614).

### 4.3. Peripheral Blood Mononuclear Cell Cultures

Experiments using peripheral blood mononuclear cells (PBMCs) were conducted according to the guidelines of the Declaration of Helsinki and approved by the Collegium Medicum of Nicolaus Copernicus University Bioethical Commission (KB 39/2019). Informed consent for participation was obtained from all subjects involved in the study. After obtaining informed consent, 18 mL of fresh blood was collected into two heparin sodium blood tubes (Medlab Products, Raszyn, Poland) from four healthy donors (aged 22–56 years) at the Occupational Medicine Clinic located in Dr. Antoni Jurasz University Hospital in Bydgoszcz, Poland. PBMCs were isolated by density gradient centrifugation (Lymphosep, BioWest, Nuaille, France). After PBMC isolation, the cell count and viability were determined using 0.4% trypan blue stain (Logos Biosystems, Gyeonggi-do, Republic of Korea) and an automated cell counter (LUNA II, Logos Biosystems, Gyeonggi-do, Republic of Korea). The cell viability was greater than 90%. For all experiments, freshly isolated PBMCs (1.0–1.5 × 10^6^ cells/mL) were cultured with compounds **2a**–**2f** and racemic ibuprofen (IBU) (Sigma Aldrich, Burlington, MA, USA) in RPMI 1640 medium (Biomed Lublin, Lublin, Poland) in the presence of 5% heat-inactivated fetal bovine serum (FBS) (Euroclone, Pero, Milan, Italy). All studied compounds and IBU were initially dissolved in DMSO (Sigma Aldrich, Burlington, MA, USA), and then in the culture medium to achieve concentrations of 10, 50, and 100 µg/mL.

### 4.4. Cell Toxicity Analysis Protocol

**Figure 10:**
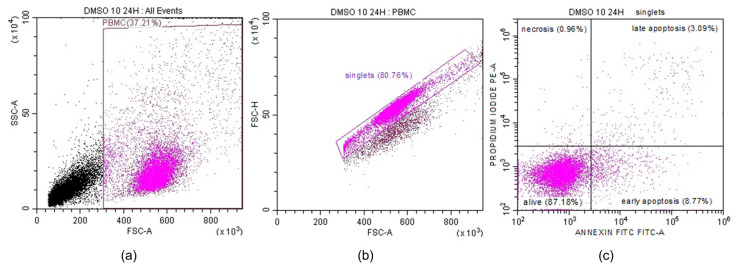
Flow cytometry gating strategy for identifying live, early apoptotic, late apoptotic, and necrotic cells in PBMC culture: (**a**) PBMCs were gated using a Forward Scatter Area (FSC-A)/Side Scatter Area (SSC-A) dot plot. (**b**) Singlet PBMCs were gated using FSC-A and Forward Scatter Height (FSC-H). (**c**) Cells were analyzed using an annexin V/propidium iodide (PI) dot plot. The identified regions are as follows: necrotic cells (annexin V−/PI+), late apoptotic cells (annexin V+/PI+), early apoptotic cells (annexin V+/PI−), live cells (annexin V−/PI−). The gating strategy shown represents PBMCs stimulated with 10 µL of DMSO. The same gating strategy applied to all samples.

The toxic effects of the studied compounds were assessed in PBMC culture using flow cytometry. PBMCs were incubated with compounds **2a**–**2f** at concentrations of 10, 50, and 100 µg/mL in polypropylene, round-bottom tubes (FALCON, Corning Science Mexico, Tamaulipas, Mexico) for 24 h at 37 °C and in a 5% CO_2_ condition. Control cultures included PBMCs only or PBMCs with DMSO (0.05–0.50%, corresponding to the solvent concentrations in the cultures with the compounds), or PBMCs with DMSO and IBU (at concentrations of 10, 50, and 100 µg/mL). After incubation, apoptosis was assessed using annexin V-FITC and propidium iodide (PI) double staining (Annexin V Apoptosis Detection Kit I, BD Pharmingen, San Diego, CA, USA). The cells were then analyzed using a CytoFLEX flow cytometer (Beckman Coulter, Suzhou, China). Flow cytometry acquisition and analysis were performed on at least 10,000 acquired events using CytExpert 2.3 software (Beckman Coulter, Suzhou, China). The flow cytometric gating strategy is shown in Figure 10.

### 4.5. Lymphocyte Proliferation Assay Protocol

The antiproliferative effects of the studied compounds were assessed in BD Horizon Violet Proliferation Dye 450 (VPD450, BD Pharmingen)-stained PBMC cultures using flow cytometry. Freshly isolated PBMCs (10–20 × 10^6^ cells/mL of PBS) were labelled with 1uM VPD450 for 11 min at 37 °C. The labelling reaction was stopped by medium containing 10% FBS, and the cells were then re-suspended to a concentration of 1 × 10^6^ cells/mL in RPMI 1640 supplemented with 5% FBS.

**Figure 11:**
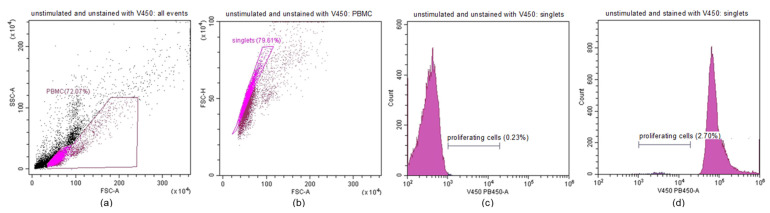
Representative flow cytometry gating strategy identifying proliferating lymphocytes in PBMCs: (**a**) PBMCs were gated on a Forward Scatter Area (FSC-A)/Side Scatter Area (SSC-A) dot plot. (**b**) Singlet PBMCs were gated on FSA-A and FSC Height (FSA-H). (**c**) Proliferating cells in non-VPD450-labelled PBMCs were identified on a VPD450 histogram. (**d**) Proliferating cells in VPD450-labelled PBMCs were identified on a VPD450 histogram. The represented gating strategy refers to unstimulated non-VPD450- and VPD450-labelled PBMCs. The same gating strategy was applied to all culture samples.

500 µL of VPD450-stained PBMCs were cultured in conical polypropylene tubes (Googlab Scientific, Rokocin, Poland) for 72 h at 37 °C in a 5% CO_2_ atmosphere. The cultures were stimulated with phytohemagglutinin (PHA) (Sigma Aldrich, Burlington, MA, USA) (2 µL) and increasing concentration of compounds **2a**–**2f** (10, 50, and 100 μg/mL) dissolved in DMSO. Control samples included PHA alone (positive control), PHA and DMSO, or PHA and IBU. After incubation, the culture tubes were centrifuged at 400× *g* at room temperature (RT) for 5 min, washed once with PBS, and 10,000 cells per sample were acquired on a CytoFLEX flow cytometer (Beckman Coulter Life Sciences, Brea, CA, USA). Data analysis was performed using CytExpert 2.3 software. The flow cytometric gating strategy is shown in Figure 11.

### 4.6. Cytokine Assay Protocol

PBMCs were stimulated with lipopolysaccharide (LPS) from *E. coli* serotype O55:B5 (Sigma-Aldrich, Burlington, MA, USA) at a concentration of 1 μg/mL, along with increasing concentrations of compounds **2a**–**2f**, for 24 h in 24-well polypropylene, non-adherent plates (CytoGen, Zgierz, Poland). Control PBMC cultures contained either medium alone, LPS and DMSO, or LPS and IBU. After incubation, samples were centrifuged at room temperature for 5 min at a speed of 400× *g*. The culture supernatants were collected into 1.5 mL tubes (Nest Biotechnology, Wuxi, Jiangsu, China) and frozen at −30 °C. After thawing, the supernatants were used to assess the concentrations of IL-1β, IL-6, TNF-α, and IL-10 using the enzyme-linked immunosorbent method (ELISA) and BD OptEIA Set Human ELISA kits (Becton Dickinson, Franklin Lakes, NJ, USA). The standard concentration ranges were as follows: IL-1β: 3,9–250 pg/mL, IL-6: 4,7–300 pg/mL, IL-10 and TNF-α: 7,8–500 pg/mL. The samples were analyzed with iEMS Reader MF (Labsystems, Helsinki, Finland), and cytokine concentrations were calculated using Ascent 2.4 software (Coimbatore, India).

### 4.7. Antimicrobial Activity Assay

The antimicrobial activity of derivatives **2a**–**2f** was tested against bacterial strains derived from the American Type Culture Collection (ATCC), including Gram-negative bacteria *Escherichia coli* ATCC 25922, *Yersinia enterocolitica* O3, and *Klebsiella pneumoniae* ATCC 700603; Gram-positive bacterium *Staphylococcus aureus* ATCC 25923; pathogenic yeast *Candida albicans* ATCC 90,028; and *Mycobacterium smegmatis,* an isolate deposited in the collection of the Department of Genetics and Microbiology, Maria Curie-Skłodowska University.

The minimum inhibitory concentration (MIC) value was determined in vitro using the microdilution method in sterile 96-well microplates, following the guidelines of the Clinical and Laboratory Standards Institute (CLSI) [20]. The tested samples were dissolved in DMSO at a concentration of 10.24 mg/mL, then diluted ten-fold, followed by two-fold serial dilutions (ranging from 512 to 0.5 μg/mL) in Mueller–Hinton (MH) broth.

### 4.8. Statistical Analysis

Data were analyzed and visualized using GraphPad software (ver. 10.2.3, Dotmatics, Boston, MA, USA). All *p*-values were calculated using the nonparametric Mann–Whitney U test.

## 5. Conclusions

Six new compounds, **2a**–**2f**, containing a cyclohex-1-ene-1-carboxylic acid moiety, were designed, synthesized, and studied for biological activity. Derivatives **2a** and **2c**–**2f** showed dose-dependent antiproliferative activity against PBMCs, comparable to or stronger than IBU. Compounds **2a**, **2d**, and **2f** almost completely inhibited lymphocyte proliferation at the highest concentration. Moreover, derivative **2d**, at the highest dose, elevated TNF-α levels, suggesting potential anticancer activity. Compound **2f** consistently reduced TNF-α production across all doses, indicating its potential as an anti-inflammatory drug. Compound **2b** at a concentration of 100 µg/mL strongly inhibited IL-6, TNF-α, and IL-10 release and exhibited antibacterial activity against *Y. eneterocolitica,* but its toxicity may limit its therapeutic use. Derivative **2c** demonstrated the strongest antibacterial activity against *M. smegmatis* and *S. aureus*. It is worth noting that the antibacterial activity of compound **2c** was observed at a concentration of 64 µg/mL, where it was non-toxic to PBMCs.

Some structure–activity relationships were identified for the studied compounds. For example, the 2-pyridyl substituent at the R^1^ position enhances antiproliferative and antibacterial potency, while the presence of two 2-pyridyl substituents is essential for cytokine release inhibition. Additionally, the 4-NO_2_-phenyl substituent may increase antiproliferative activity, while the 4-CH_3_-phenyl substituent may enhance both antiproliferative and antibacterial effects. The presence of an unsaturated double bond in the cyclohexene ring may further boost antiproliferative activity and antibacterial efficacy against Gram-negative bacterial strains, such as *Y. enterocolitica*. These findings could guide the development of new drug candidates among amidrazone derivatives.

## Figures and Tables

**Table 1:** Minimum inhibitory concentration (MIC) values (µg/mL) of compounds **2a**–**2f** against tested bacterial and fungal strains compared to ampicillin (Amp) and fluconazole (Flu).

Strain	MIC Values (µg/mL)							
	2a	2b	2c	2d	2e	2f	Amp	Flu
*Mycobacterium smegmatis*	64	512	64	512	512	>512	16	-
*Staphylococcus aureus* ATCC 25923	256	256	64	512	512	>512	0.25	-
*Escherichia coli* ATCC 25922	512	256	512	512	>512	512	8	-
*Yersinia enterocolitica* O3	512	64	512	512	512	128	16	-
*Klebsiella pneumoniae* ATCC 700603	512	256	256	512	512	>512	>256	-
*Candida albicans* ATCC 90028	512	>512	512	512	512	256	-	0.25

## Data Availability

Data are available from the authors.

## Supplementary Materials

The following supporting information can be downloaded at https://www.mdpi.com/article/10.3390/molecules30081853/s1: Figure S1. 1H NMR spectrum of compound **2a**. Figure S2. ^1^H NMR spectrum of compound **2b**. Figure S3. ^1^H NMR spectrum of compound **2c**. Figure S4. ^1^H NMR spectrum of compound **2d**. Figure S5. ^1^H NMR spectrum of compound **2e**. Figure S6. ^1^H NMR spectrum of compound **2f**. Figure S7. ^13^C NMR spectrum of compound **2a**. Figure S8. ^13^C NMR spectrum of compound **2b**. Figure S9. ^13^C NMR spectrum of compound **2c**. Figure S10. ^13^C NMR spectrum of compound **2d**. Figure S11. ^13^C NMR spectrum of compound **2e**. Figure S12. ^13^C NMR spectrum of compound **2f**. Figure S13. Representative flow cytometric analysis of alive, early apoptotic, late apoptotic, and necrotic cells in PBMC cultures stimulated with different doses of DMSO (top row), IBU (middle row), and compound **2a** (bottom row). Figure S14. Representative flow cytometry analysis of proliferating lymphocytes in PHA-stimulated VPD-450-labelled PBMC cultures exposed to different doses of DMSO (top row), IBU (middle row), and compound **2a** (bottom row). Table S1. Toxicity of compounds **2a**–**2f** toward PBMCs (24h). Figure S15. The effect of compounds **2a**–**2f** on IL-1β production in 72 h PBMC cultures stimulated with lipopolysaccharide (LPS).

## Abbreviations

ATCC	American Type Culture Collection
DAMP	danger/damage-associated molecular pattern
DMSO	dimethyl sulfoxide
IBU	ibuprofen
IL	interleukin
LPS	lipopolysaccharide
MIC	minimal inhibitory concentration
NET	neutrophil extracellular traps
PAMP	pathogen-associated molecular pattern
PBMCs	peripheral blood mononuclear cells
PHA	phytohemagglutinin
TNF-α	tumor necrosis factor-alpha
