# Supplementary material for: Synthesis, Evaluation of Biological Activity, and Structure–Activity Relationships of New Amidrazone Derivatives Containing Cyclohex-1-ene-1-Carboxylic Acid
Source: Molecules. 2025 Apr 21;30(8):1853. doi: 10.3390/molecules30081853 (PMC12029444; doi:10.3390/molecules30081853)
Supplement: Supplementary file 1 [file molecules-30-01853-s001.zip › molecules-3571035-supplementary.pdf]

Synthesis, Evaluation of Biological Activity, and Structure–Activity Relationships of New Amidrazone Derivatives Containing Cyclohex-1-ene-1-Carboxylic Acid

Renata Paprocka [1,*], Jolanta Kutkowska [2], Ewelina Paczkowska [3], Godwin Munroe Mwaura [4], Andrzej Eljaszewicz [5] and Anna Helmin-Basa [3]

1 Department of Organic Chemistry, Faculty of Pharmacy, Collegium Medicum in Bydgoszcz, Nicolaus Copernicus University in Toruń, Jurasza Str. 2, 85-089 Bydgoszcz, Poland
2 Department of Genetics and Microbiology, Institute of Biological Sciences, Maria Curie-Skłodowska University, Akademicka Str. 19, 20-033 Lublin, Poland; jolanta.kutkowska@mail.umcs.pl
3 Department of Immunology, Faculty of Pharmacy, Collegium Medicum in Bydgoszcz, Nicolaus Copernicus University in Toruń, M. Curie-Sklodowska Str. 9, 85-094 Bydgoszcz, Poland; e.paczkowska97@gmail.com (E.P.); a.helmin-basa@cm.umk.pl (A.H.-B.)
4 Department of Pharmaceutical Chemistry, Pharmaceutics and Pharmacognosy, Faculty of Health Sciences, University of Nairobi, KNH, Nairobi P.O. Box 2149-00202, Kenya; godwinmunroe1@gmail.com
5 Centre of Regenerative Medicine, Medical University of Bialystok, Waszyngtona 15 B, 15-269 Bialystok, Poland; andrzej.eljaszewicz@umb.edu.pl
* Correspondence: renata.bursa@cm.umk.pl

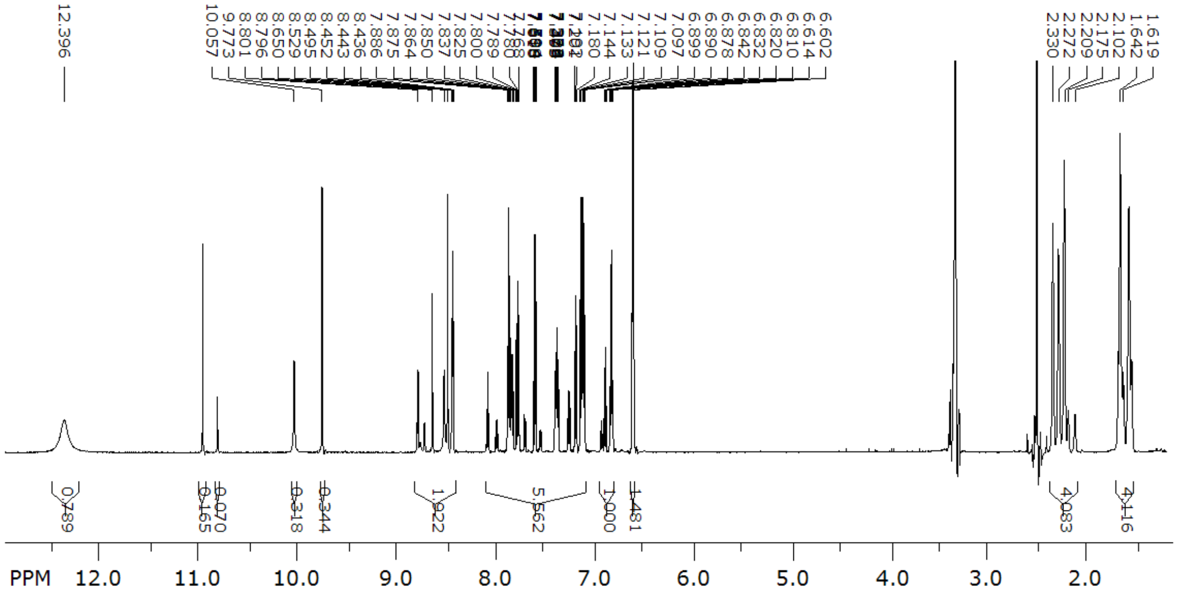

Figure S1. ^{1}H NMR spectrum of compound **2a**.

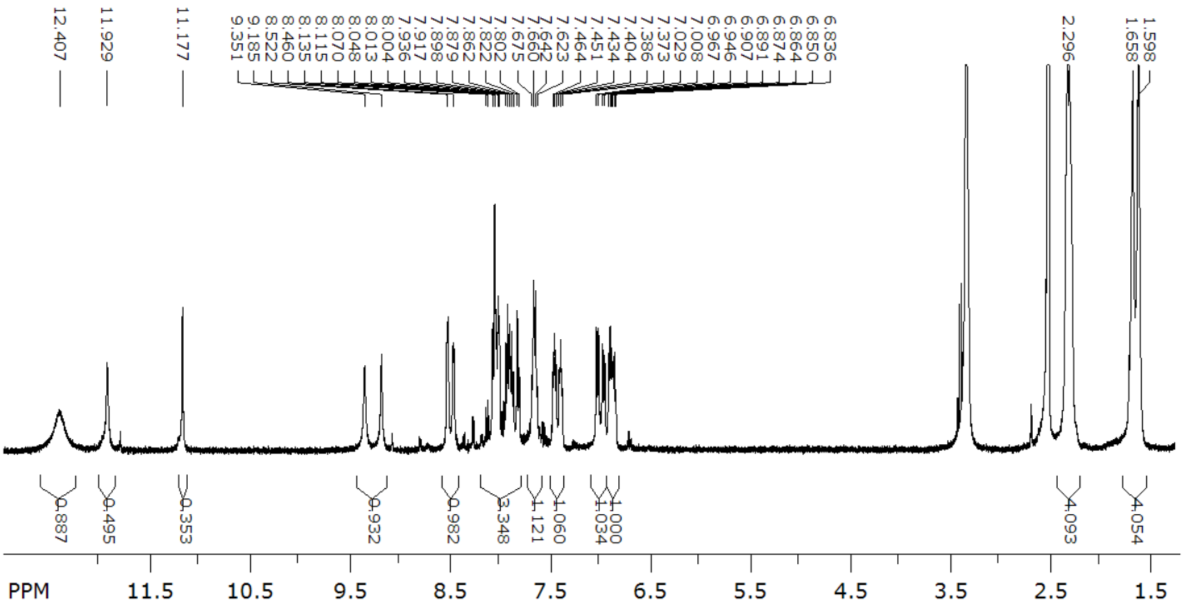

Figure S2. ^{1}H NMR spectrum of compound **2b**.

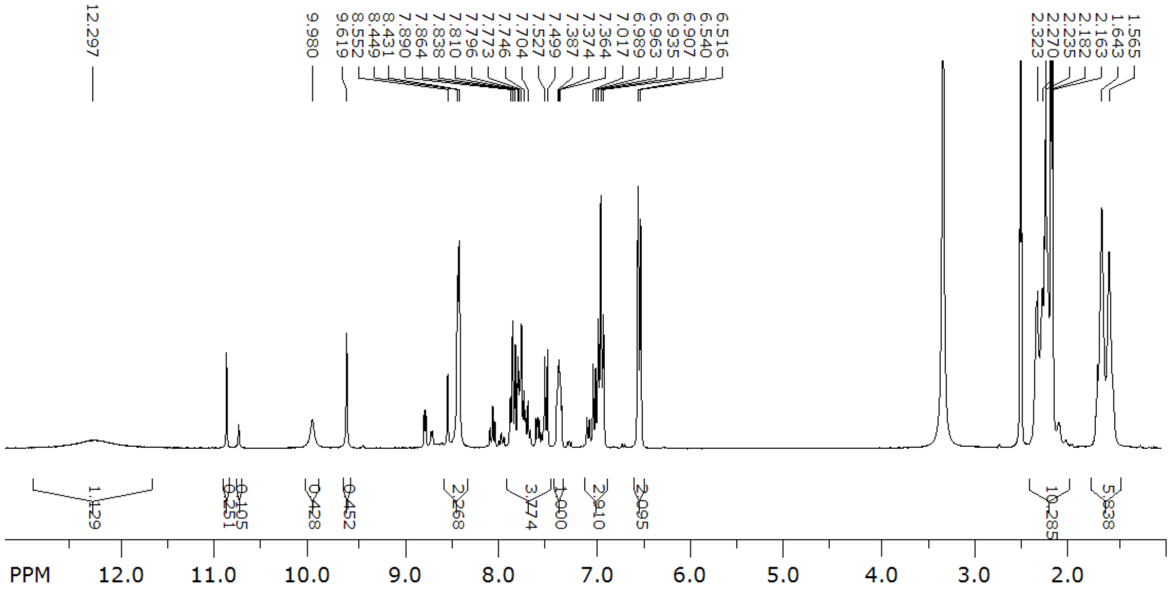

Figure S3. ^{1}H NMR spectrum of compound **2c**.

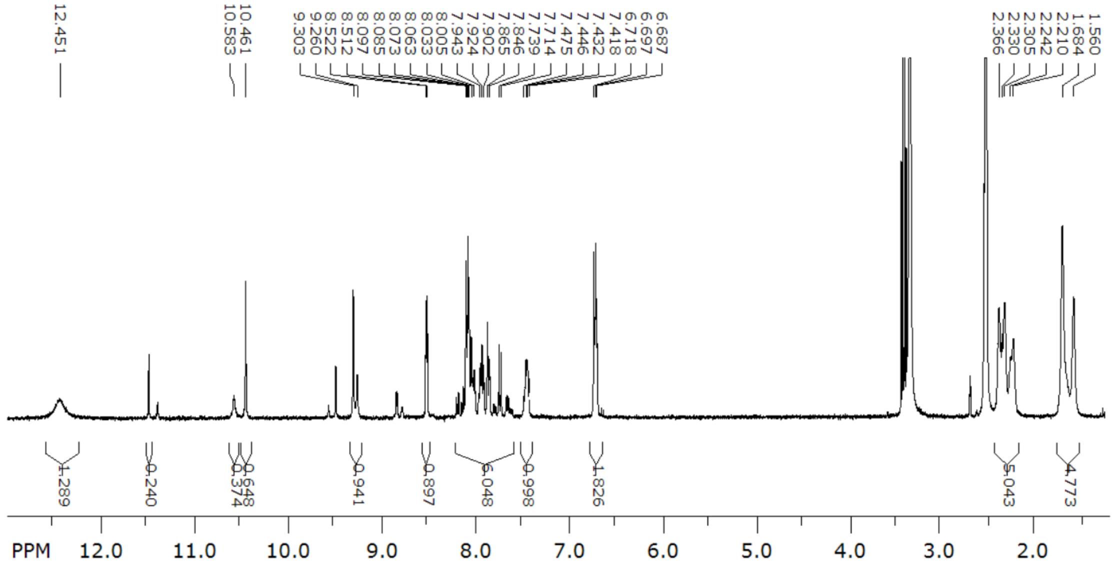

Figure S4. ^{1}H NMR spectrum of compound **2d**.

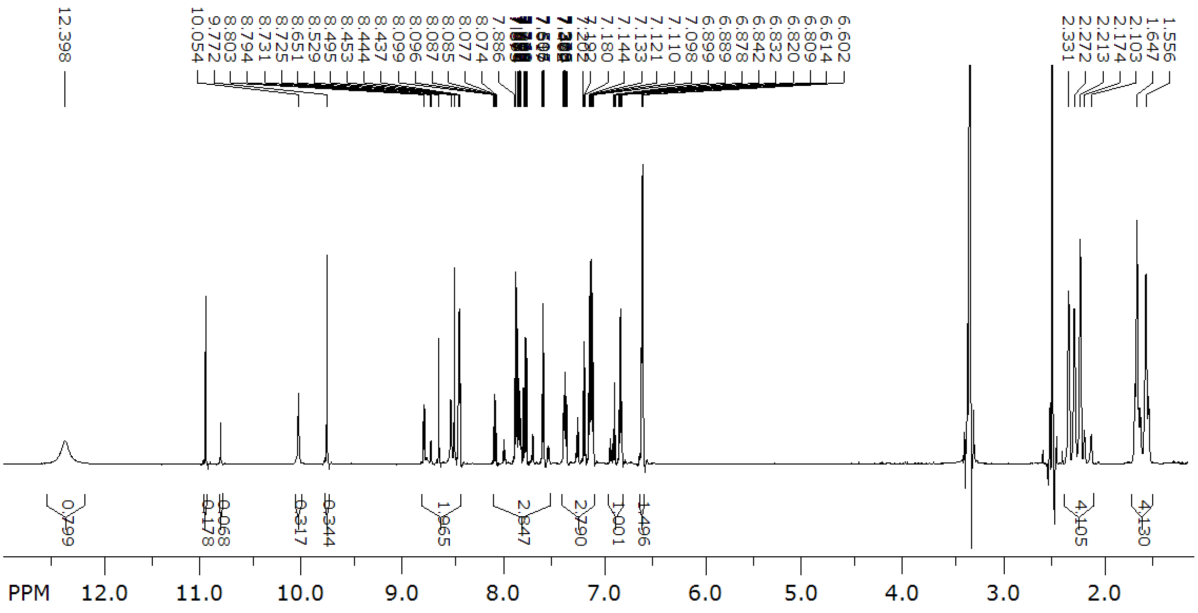

Figure S5. ^{1}H NMR spectrum of compound **2e**.

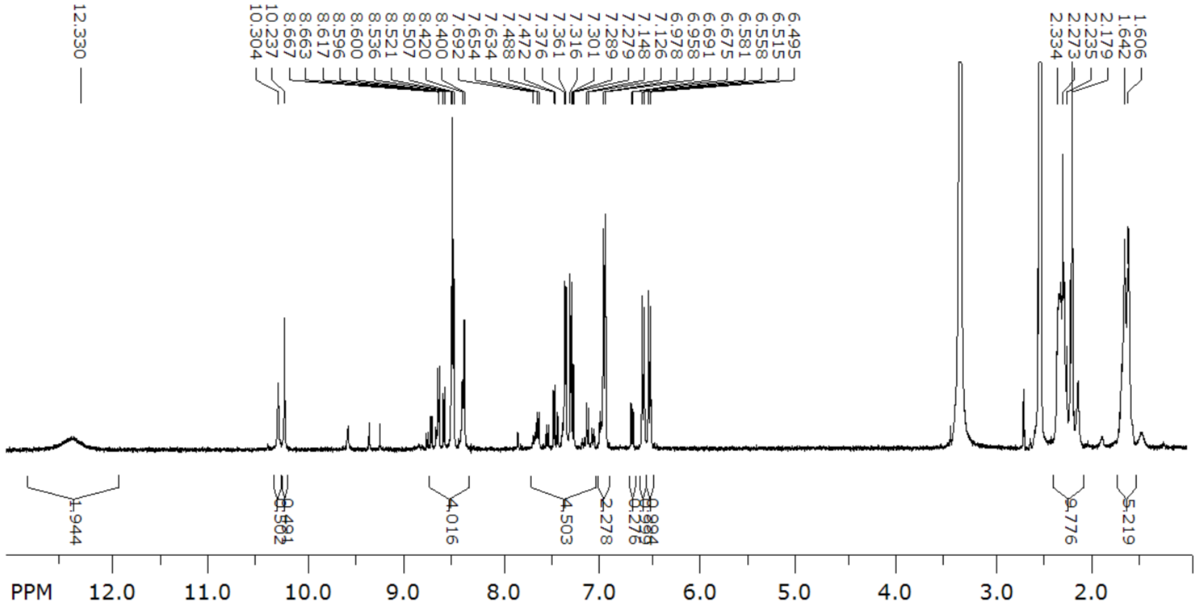

Figure S6. ^{1}H NMR spectrum of compound **2f**.

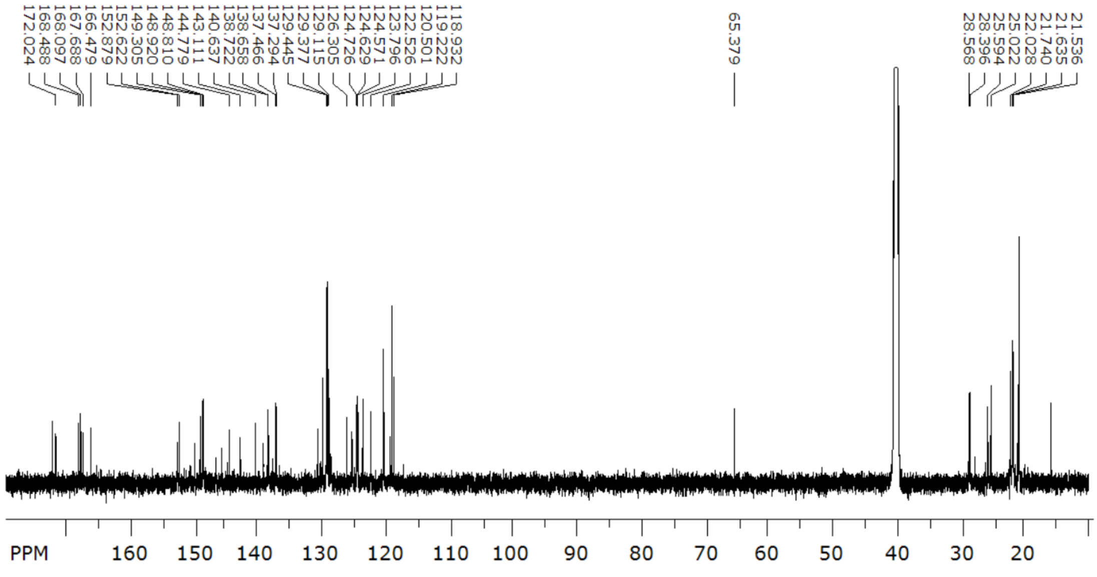

Figure S7. ^{13}C NMR spectrum of compound **2a**.

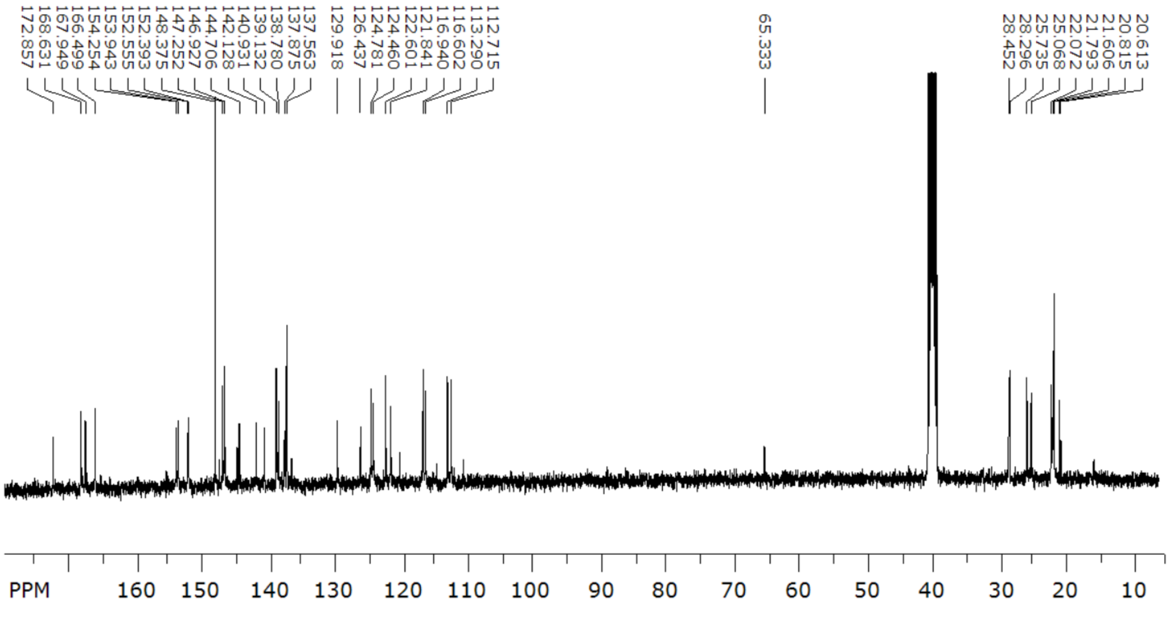

Figure S8. ^{13}C NMR spectrum of compound **2b**.

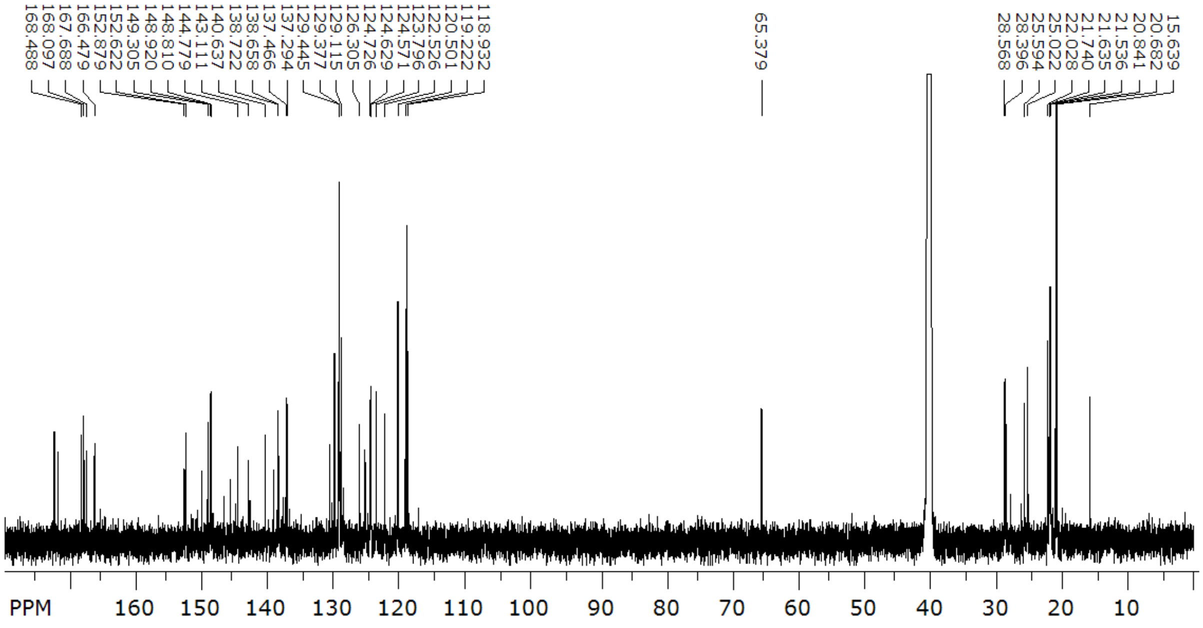

Figure S9. ^{13}C NMR spectrum of compound **2c**.

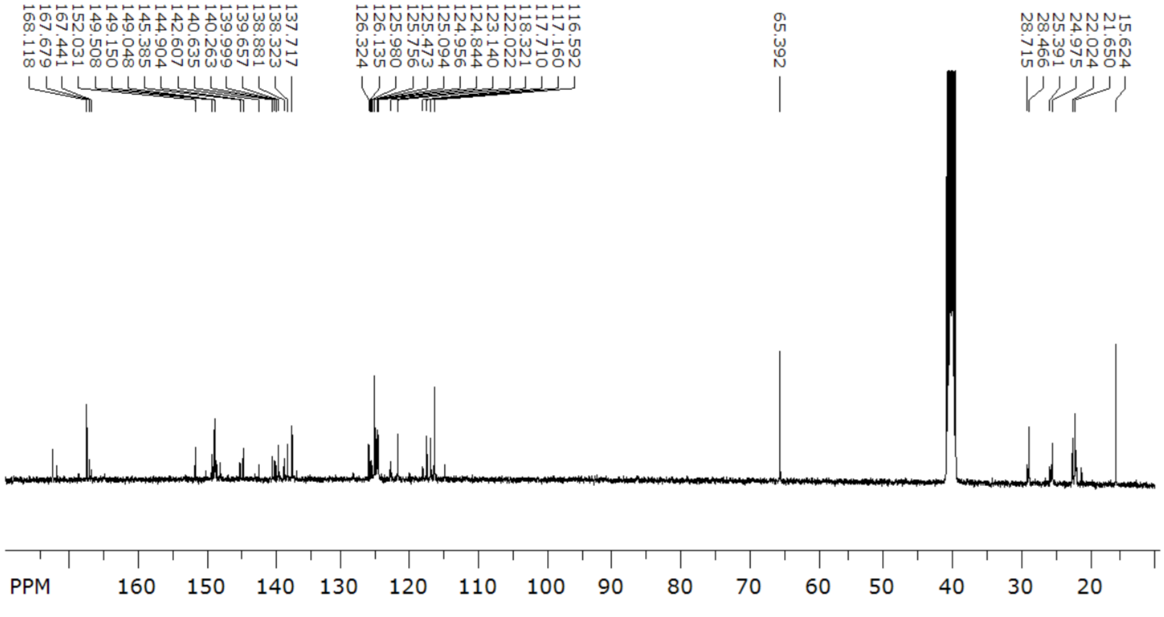

Figure S10. ^{13}C NMR spectrum of compound **2d**.

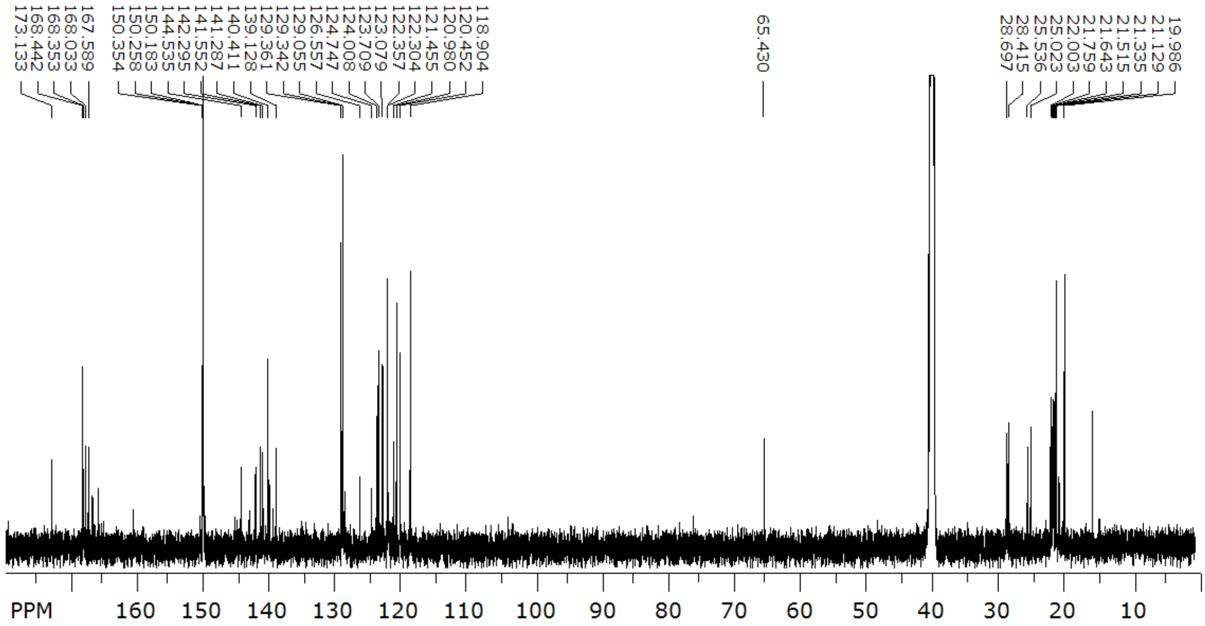

Figure S11. ^{13}C NMR spectrum of compound **2e**.

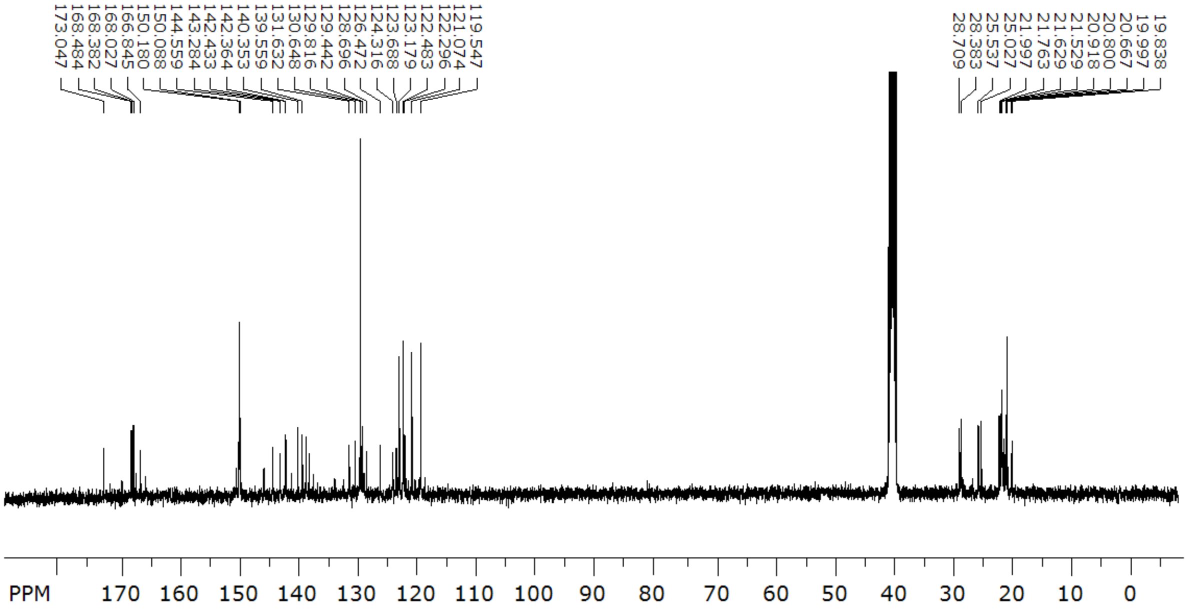

Figure S12. ^{13}C NMR spectrum of compound **2f**.

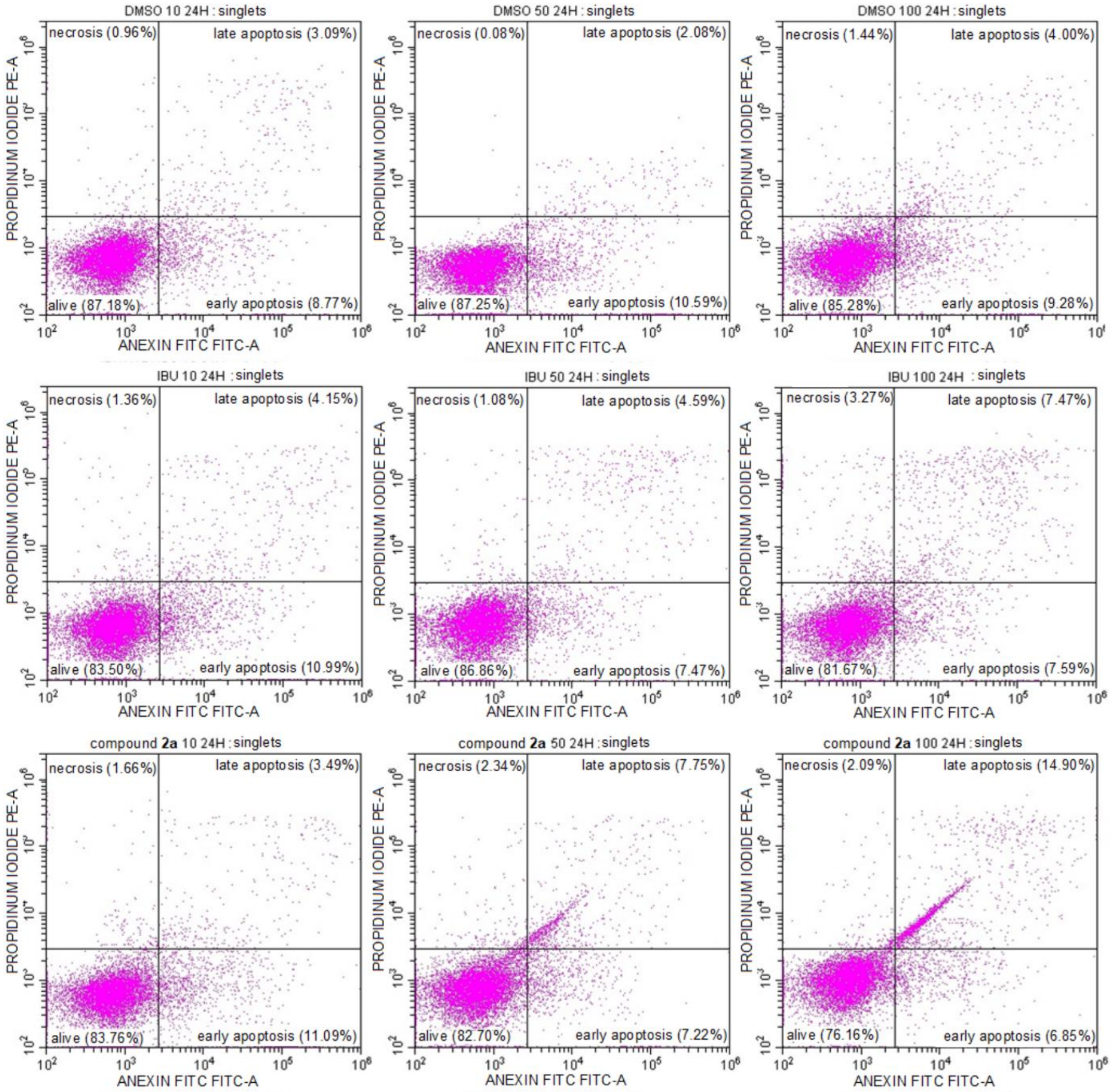

Figure S13. Representative flow cytometric analysis of alive, early apoptotic, last apoptotic and necrotic cells in PBMC cultures stimulated with different doses of DMSO (**top row**), IBU (**middle row**) and compound **2a** (**bottom row**).

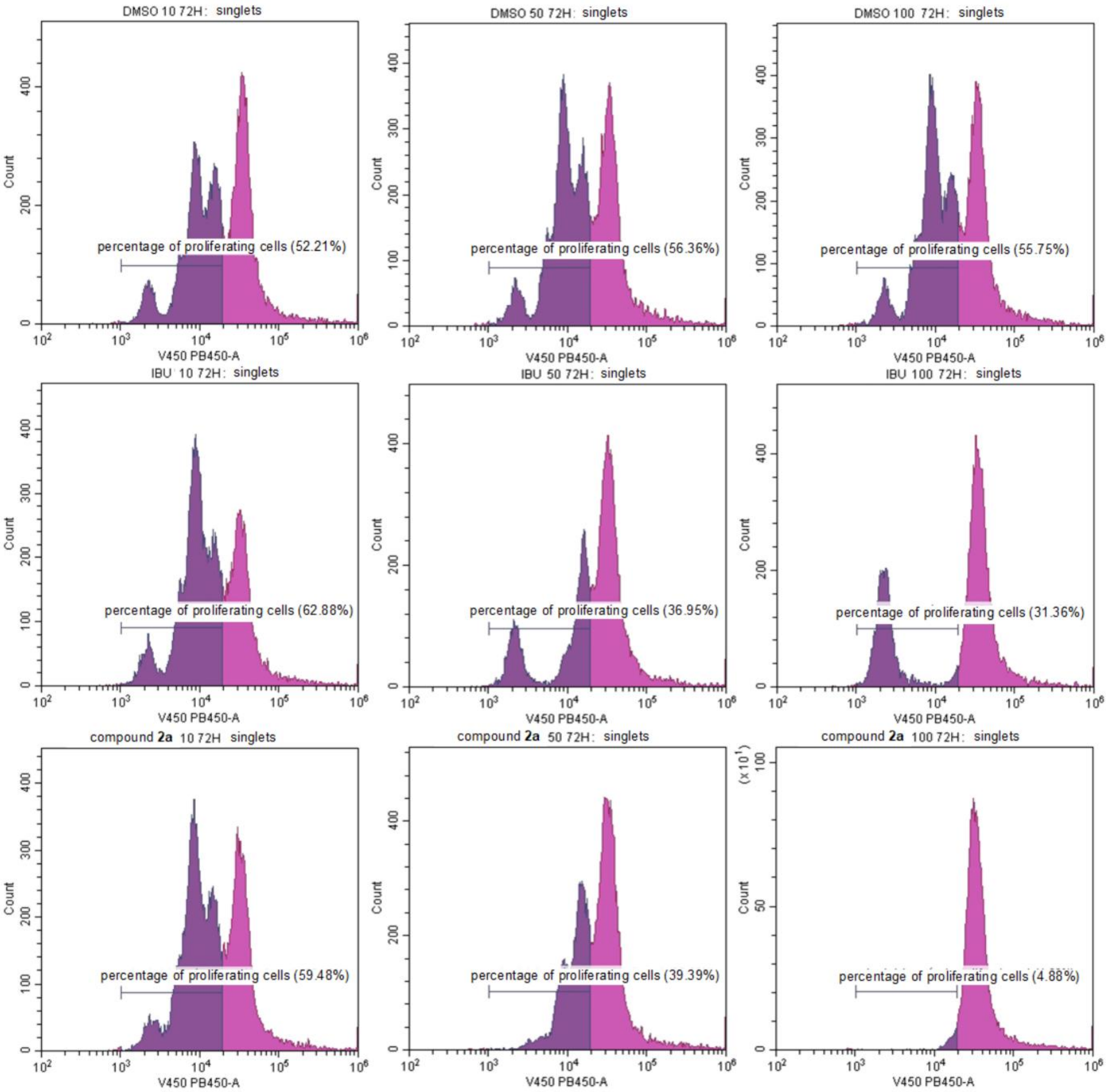

Figure S14. Representative flow cytometry analysis of proliferating lymphocytes in PHA-stimulated VPD-450 labelled PBMC cultures exposed to different doses of DMSO (**top row**), IBU (**middle row**) and compound **2a** (**bottom row**).

Table S1. Toxicity of compounds **2a-2f** towards PBMCs (24h)

	alive %	early apoptosis %	late apoptosis %	necrosis %
(-)	86.82%	9.82%	2.51%	0.85%
DMSO 0.05%	87.18%	8.77%	3.09%	0.96%
DMSO 0.025%	87.25%	10.59%	2.08%	0.08%
DMSO 0.5%	85.28%	9.28%	4.00%	1.44%
IBU 10 µg/ml	83.50%	10.99%	4.15%	1.36%
IBU 50 µg/ml	86.86%	7.47%	4.59%	1.08%
IBU 100 µg/ml	81.67%	7.59%	7.47%	3.27%
2a 10 µg/ml	83.76%	11.09%	3.49%	1.66%
2a 50 µg/ml	82.70%	7.22%	7.75%	2.34%
2a 100 µg/ml	76.16%	6.85%	14.90%	2.09%
2b 10 µg/ml	84.69%	9.13%	4.34%	1.84%
2b 50 µg/ml	72.66%	13.63%	12.50%	1.22%
2b 100 µg/ml	35.52%	23.66%	35.51%	5.31%
2c 10 µg/ml	84.83%	10.43%	3.79%	0.95%
2c 50 µg/ml	82.56%	7.73%	7.73%	1.98%
2c 100 µg/ml	75.51%	9.68%	11.02%	3.79%
2d 10 µg/ml	83.29%	11.54%	4.23%	0.93%
2d 50 µg/ml	72.00%	10.34%	15.66%	2.00%
2d 100 µg/ml	64.30%	8.99%	22.57%	4.14%
2e 10 µg/ml	83.19%	11.34%	4.54%	0.92%
2e 50 µg/ml	81.61%	9.43%	7.58%	1.38%
2e 100 µg/ml	75.90%	7.61%	13.51%	2.98%
2f 10 µg/ml	83.80%	8.80%	6.10%	1.30%
2f 50 µg/ml	70.37%	7.07%	20.13%	2.43%
2f 100 µg/ml	63.12%	6.64%	27.23%	3.02%

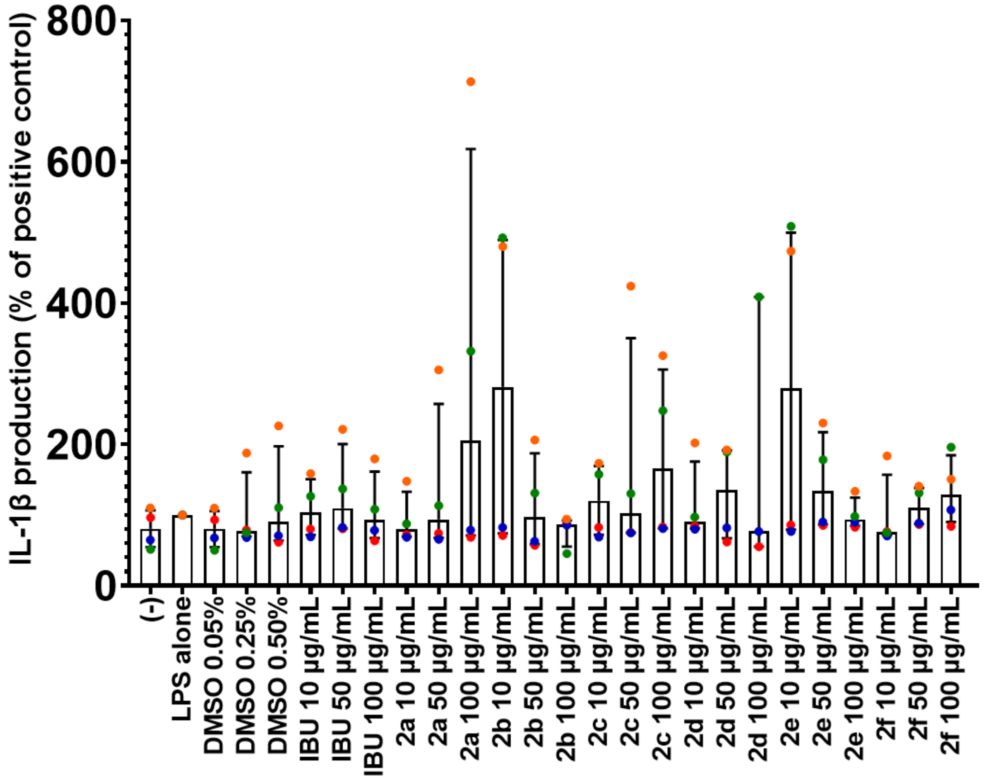

Figure S15. The effect of compounds **2a-2f** on IL-1β production in 72 h PBMC culture stimulated with lipopolysaccharide (LPS). Results (4 independent experiments, median and Q1-Q3 range) are presented as a percentage of positive control (cultures stimulated with LPS alone). Dots in different colors indicate the results of independent experiments. No significant differences were observed (Mann-Whitney U test).
